# Polymerization of dietary fructans differentially affects interactions among intestinal microbiota of colitis mice

**DOI:** 10.1093/ismejo/wrae262

**Published:** 2025-01-02

**Authors:** Yaqin Xiao, Qianyun Zhao, Dawei Ni, Xiaoqi Zhang, Wei Hao, Qin Yuan, Wei Xu, Wanmeng Mu, Dingtao Wu, Xu Wu, Shengpeng Wang

**Affiliations:** State Key Laboratory of Quality Research in Chinese Medicine, Institute of Chinese Medical Sciences, University of Macau, Avenida da Universidade, Taipa, Macao 999078, China; Laboratory of Molecular Pharmacology, Department of Pharmacology, Southwest Medical University, Xianglin Road, Longmatan District, Luzhou, Sichuan 646000, China; State Key Laboratory of Food Science and Resources, Jiangnan University, Lihu Avenue, Wuxi, Jiangsu 214122, China; State Key Laboratory of Food Science and Resources, Jiangnan University, Lihu Avenue, Wuxi, Jiangsu 214122, China; State Key Laboratory of Quality Research in Chinese Medicine, Institute of Chinese Medical Sciences, University of Macau, Avenida da Universidade, Taipa, Macao 999078, China; State Key Laboratory of Quality Research in Chinese Medicine, Institute of Chinese Medical Sciences, University of Macau, Avenida da Universidade, Taipa, Macao 999078, China; State Key Laboratory of Food Science and Resources, Jiangnan University, Lihu Avenue, Wuxi, Jiangsu 214122, China; State Key Laboratory of Food Science and Resources, Jiangnan University, Lihu Avenue, Wuxi, Jiangsu 214122, China; Key Laboratory of Coarse Cereal Processing, Ministry of Agriculture and Rural Affairs, Sichuan Engineering & Technology Research Center of Coarse Cereal Industrialization, School of Food and Biological Engineering, Chengdu University, Chengluo Avenue, Chengdu, Sichuan 616106, China; Laboratory of Molecular Pharmacology, Department of Pharmacology, Southwest Medical University, Xianglin Road, Longmatan District, Luzhou, Sichuan 646000, China; State Key Laboratory of Quality Research in Chinese Medicine, Institute of Chinese Medical Sciences, University of Macau, Avenida da Universidade, Taipa, Macao 999078, China

**Keywords:** Fructans, Structure-activity relationship, Microbiota, Colitis, *Dubosiella*

## Abstract

The intestinal microbiota plays a critical role in maintaining human health and can be modulated by dietary interventions and lifestyle choices. Fructans, a dietary carbohydrate, are selectively utilized by the intestinal microbiota to confer health benefits. However, the specific effects of different fructan types on microbial changes and functions remain incompletely understood. Here, we investigated how the intestinal microbiota responds to fructans with varying degrees of polymerization in the context of gut dysbiosis. Both low molecular weight fructo-oligosaccharides and high molecular weight levan suppressed intestinal inflammation in a colitis mouse model, mitigating intestinal fibrosis and dysbiosis. Although both the effects of fructo-oligosaccharides and levan are microbiota-dependent, distinct modulation patterns of the intestinal microbiota were observed based on the molecular weight of the fructans. Levan had a more pronounced and persistent impact on gut microbiota compared to fructo-oligosaccharides. Levan particularly promoted the abundance of *Dubosiella newyorkensis*, which exhibited preventive effects against colitis. Our findings highlight the importance of polymerization levels of dietary fructans in microbiota alterations and identify *Dubosiella newyorkensis* as a potential probiotic for treating inflammatory diseases.

## Graphical Abstract

**Graphical Abstract:**
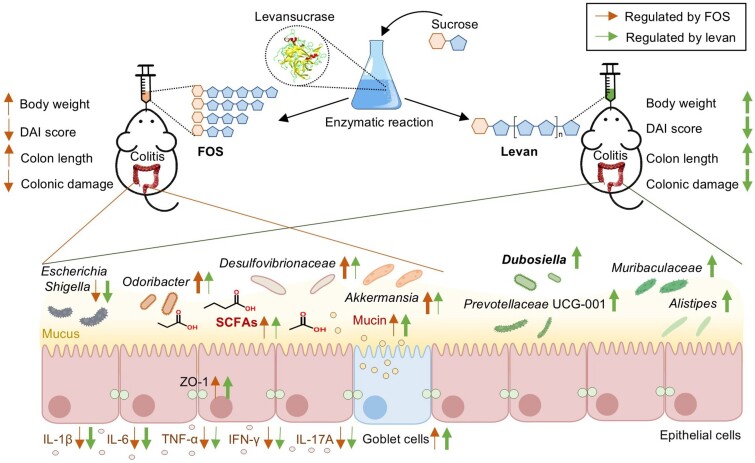

## Introduction

Fructans naturally exist in various plants, bacteria, archaea, and certain fungi [1], and are abundant in foods such as wheat products and vegetables including garlic, onion, artichoke, and asparagus [2]. Due to structural variations in their glycosidic bonds, molecular weights, and branching degrees, different sources of fructans exhibit distinct physicochemical properties and biological activities. Lacking the necessary hydrolytic enzymes [3], fructans are not absorbed in the upper gastrointestinal tract and instead serve as a major source of carbon and energy for the distal gut microbial community [4]. Consequently, fructans exhibit properties of low glycemic index, low-calorie content, and a prebiotic effect [5]. These health benefits make fructans potential functional ingredients for individuals with obesity, diabetes, or gastrointestinal disorders.

Mammals are born colonized by microbes from the environment, establishing a mutually beneficial microbial ecosystem [6, 7]. These microbes significantly shape host metabolism and immune functions, which is indispensable in understanding how environmental factors and behavior influence human biology [8, 9]. Phylogenetically diverse organisms that inhabit the same environment may exhibit convergent gene content as they adapt to stress events, particularly for functional genes crucial for survival [10]. The convergence of glycoside hydrolases and glycosyltransferases in bacteria and archaea inhabiting within the human gut underscores the significance of carbohydrate metabolism as a core function encoded within the microbiome [11]. Fructans can be utilized by a diverse array of gut bacteria [12, 13], and identifying changes in phyla or taxa can offer valuable insights into the effects of dietary interventions on gut homeostasis. Therefore, variations in the type and degree of polymerization result in differences in the composition and function of the microbial community, which have significant implications for personalized nutrition and the optimization of microbial functionality and interactions with the host.

Studies employing in vitro fermentation of human gut microbiota have demonstrated that the prebiotic activity of inulin-type fructans is contingent upon the degree of polymerization [14, 15]. A higher degree of polymerization leads to a slower fermentation rate and enhances prebiotic potency. Furthermore, higher degrees of polymerization confer greater benefits to the microbial community in both the proximal and distal colon regions. Due to the limited availability of levan-type fructans with different degrees of polymerization in the market, they have not been extensively studied. Levansucrase, a member of the glycoside hydrolase family 68, is widely distributed among both Gram-positive and Gram-negative species [16]. Levansucrase cleaves sucrose into one molecule of glucose and a covalent enzyme-fructosyl intermediate, which subsequently undergoes transfructosylation to synthesize levan-type fructans. The polymerization level of levan is highly dependent on the source of levansucrase, a property that facilitates the customization of products to meet specific research purposes.

Ulcerative colitis is a disease closely linked to alterations in the human microbiome [17]. The gut microbiota of individuals with colitis is often characterized by fragility and susceptibility to reshaping, making it a suitable subject for understanding how polysaccharides influence the gut microbiota [18]. Here, we employed a well-established colitis mouse model induced by dextran sulfate sodium (DSS) to provoke intestinal inflammation and gut microbiota dysbiosis, thereby evaluating the effect of dietary intervention on disease progression [19, 20]. By comparing low molecular weight fructo-oligosaccharides (FOS) and high molecular weight levan, we sought to assess the impact of polymerization degree on reshaping the gut microbiota by fructans.

## Materials and methods

### Levansucrase expression

The levansucrase gene (EC 2.4.1.10, GenBank AGE89831.1) from *Pseudomonas orientalis* was cloned into a pET-22b (+) expression vector modified to encode an N-terminal His6-SUMO tag. The recombinant plasmid was transformed into competent *Escherichia coli* BL21(DE3) cells (Promega, Madison, WI USA) and cultured in glass shake flasks containing 2 L of lysogenic broth (100 mg L^−1^ of ampicillin) with shaking (200 rpm min^−1^) at 37°C. Upon reaching an optical density (OD600) of 0.6, isopropylthiogalactoside (Cat. #I6758, Sigma Aldrich) was added to the medium to achieve a final concentration of 0.5 mM. Following 6 h of fermentation at 28°C with continuous shaking, the cells were harvested by centrifugation at 8000 g for 5 min. The cell pellets were subsequently washed once with 0.01 M phosphate-buffered saline (PBS), centrifuged again, and stored at -20°C.

For detailed preparation methods including molecular weight determination and structure determination of levan and FOS, please refer to the supplementary information.

### Animals experiment

Six-week-old C57BL/6 J male mice were purchased from Si Pei Fu Biotechnology Co., Ltd (Beijing, China) and housed in a specific pathogen-free environment with an average temperature of 22°C and a 12 h light–dark cycle. The mice were cohoused for a week with free access to sterile drinking water and food before being randomly assigned to experimental groups. Following acclimation, the different experimental groups and administration protocols were implemented as outlined in figures, and methods in the supplementary information. At the end of the experiment, mice were anesthetized by intraperitoneal injection with 70 mg kg^−1^ sodium pentobarbital, and blood was collected by cardiac puncture. Subsequently, the mice were sacrificed by cervical dislocation, and tissues, including the heart, liver, spleen, lung, kidney, and entire colon, were collected. Fecal samples were collected and stored at −80°C for microbiome analysis. The colon length was measured, and the colon was gently washed with PBS. Three 0.5 cm midsections of the colon were taken from each group and preserved in 4% paraformaldehyde for histological evaluation and immunofluorescence staining. The remaining colon tissue samples were snap-frozen in liquid nitrogen and stored at -80°C to determine cytokine concentrations. The animal experiments were approved by the Committee on Use and Care of Animals of Southwest Medical University (Approval number: 20220817-009).

### Disease activity index (DAI)

The DAI for the DSS-induced mouse model was evaluated blindly and described from day 0 until sacrifice. The scoring system is to divide the composite score of body weight loss, stool consistency, and rectal bleeding by three. The specific scoring criteria are as follows: body weight decrease: 0 for 0-1%, 1 for 1-5%, 2 for 5-10%, 3 for 10-20%, and 4 for 20%+; stool consistency: 0 for normal, 2 for loose stools, and 4 for diarrhea; rectal bleeding: 0 for no blood, 2 for blood in feces, and 4 for gross bleeding. The composite DAI score was calculated by averaging these individual scores.

### Serum biochemical assay

Mouse blood samples were collected into 1.5 mL Eppendorf tubes, left at room temperature for 20 min, and then centrifuged at 3000 rpm for 5 min. The resulting serum was carefully transferred to new Eppendorf tubes, and protein quantification was performed using Pierce BCA Protein Assay Kits (Cat. #23225; Thermo Scientific). The levels of C-reactive protein (CRP), alkaline phosphatase (ALP), low-density lipoprotein (LDL), and lactate dehydrogenase (LDH) in the serum were determined using an automated biochemical analyzer (BS360S, Mindray).

### Histopathological examination

Colonic tissues fixed in 4% paraformaldehyde are embedded in paraffin, sectioned, and stained with hematoxylin and eosin (H&E). Images were captured using a Nikon Eclipse E100 microscope (Nikon). Colonic histological damage was assessed blindly, with scores assigned based on the following criteria [21]: 0 for normal, 1 for chronic inflammation, 2 for lamina propria neutrophils, 3 for neutrophils invade epithelium, 4 for crypt destruction, and 5 for erosions or ulcers. Three samples were taken from each group, and the average score was calculated from five fields of view for each sample.

### Enzyme-linked immunosorbent assay (ELISA)

To determine cytokine levels in colon tissues, the colon segment was homogenized in radioimmunoprecipitation assay buffer containing 1% phenylmethanesulfonyl fluoride using a Tissue Lyser II (Qiagen) at 4°C. Each sample was centrifuged for 10 min at 10 000 rpm and 4°C. The resultant supernatants were utilized for cytokine analysis and total protein quantification. Cytokine analysis was conducted using ELISA kits (IL-1*β*, Cat. #432616; IL-6, Cat. #431316; IFN-*γ*, Cat. #430816; IL-10, Cat. #431426; IL-17A, Cat. #432504; and TNF-*α*, Cat. #430916, Biolegend) and protein quantification was performed using Pierce BCA Protein Assay Kits (Cat. #23225; Thermo Scientific).

### Immunofluorescence staining

Paraffin-embedded colon tissue sections were deparaffinized and washed three times with ethanol. Antigen retrieval was performed using Ethylenediaminetetraacetic acid (EDTA, Cat. #ST1303, Beyotime) at pH 8.0. After blocking with 5% bovine serum albumin (Cat. #ST023, Beyotime) for 30 min, the primary antibody was added and incubated overnight at 4°C. After washing three times with PBS (pH 7.4), the secondary antibody (CY3-labeled goat anti-rabbit IgG) was added and incubated at room temperature for 50 min in the dark. Following further washes, slides were stained with 4′,6-diamidino-2-phenylindole (DAPI, Cat. #C1002, Beyotime) for 10 min and then washed. An autofluorescence quencher reagent B was applied for 5 minutes, then rinsed with running water for 10 minutes, and finally, the slides were sealed with an antifade reagent. Images were captured using a Nikon Eclipse C1 microscope (Nikon, Japan). DAPI has an excitation wavelength of 330–380 nm and an emission wavelength of 420 nm; the target protein exhibits an excitation wavelength of 510–560 nm and an emission wavelength of 590 nm. Detailed antibody information is provided in supplementary Table S2.

### GC–MS targeted metabolomic analysis of SCFA concentrations

Mouse feces (20 mg) were accurately weighed in 2 mL grinding tubes and mixed with 800 μL of 0.5% phosphoric acid solution containing 2-ethylbutyric acid at a concentration of 10 μg mL^−1^. The samples were frozen and ground for 3 min, followed by sonication for 10 min. The samples were then centrifuged at 4°C and 13 000 g for 15 min. Following centrifugation, 200 μL of the supernatant was transferred to a new tube and mixed with 200 μL of butanol, vortexed for 10 seconds, and then sonicated for an additional 10 min. The mixture was allowed to centrifuge again at 4°C and 13 000 g for 5 min, and the supernatant was aliquoted for GC-MS analysis.

SCFA separation was carried out using an 8890B-7000D GC/MSD GC–MS (Agilent Techologies Inc), equipped with an HP-FFAP GC column (30 m × 0.25 mm × 0.25 m, Agilent J&W Scientific). High-purity ammonia gas was used as the carrier gas at a flow rate of 1.0 mL min^−1^. The inlet temperature was set at 180°C with an injection volume of 1 μL and a split ratio of 10:1. The temperature program began with an initial temperature of 80°C, ramped to 120°C at a rate of 20°C min^−1^, followed by an increase to 160°C at a rate of 5°C min^−1^, and finally held at 220°C for 3 min. The chromatographic conditions consisted of an electron impact ion source (EI), ion source temperature set to 230°C, quadrupole temperature at 150°C, transfer line temperature at 230°C, and electron energy of 70 eV. The scan mode employed was selected ion monitoring (SIM) mode. Masshunter quantitative software (version 10.0.707.0) was used to automatically identify and integrate the ion fragments of the target SCFAs.

### 16S ribosomal RNA gene sequencing

Mouse feces were collected and immediately frozen in liquid nitrogen, then transported on dry ice to Majorbio Bio-Pharm Technology Co. Ltd. (Shanghai, China) for 16S rRNA gene sequencing. The total DNA was extracted from 100 mg of feces following the instructions of OMG-Soil DNA Kit (Omega Bio-Tek Georgi, GA, USA). The concentration and quality of the extracted DNA were measured by a NanoDrop 2000 UV–vis spectrophotometer (Thermo Scientific, Wilmington, USA). After DNA extraction, the V3-V4 variable region of the 16S rRNA gene was amplified with primer pairs 338F (5'-ACTCCTACGGGAGGCAGCAG-3′) and 806R (5'-GGACTACHVGGGTWTCTAAT-3′) by PCR. The NEXTflex Rapid DNA-Seq (Bioo Scientific) was used for library preparation, and sequencing was conducted on the NovaSeq PE250 platform (Illumina, USA). The original sequencing reads were quality-filtered using fastp software (version 0.20.0) and assembled using FLASH software (version 1.2.7). OTU clustering and chimera removal were performed using UPARSE software (version 7.1) based on a 97% similarity cutoff [22]. Taxonomic classification of each OTU representative sequence was conducted using the RDP classifier (version 2.2), aligning against the SILVA 16S rRNA gene database (version 138) with a 70% confidence threshold. The analysis of the 16S rRNA gene sequencing results were carried out utilizing the Majorbio cloud platform (www.majorbio.com). The complete list of valid sequence number for each sample is provided in supplementary Table S3.

### Strain culture

*Dubosiella newyorkensis* NYU-BL-A4 obtained from the American Type Culture Collection (TSD-64) was cultured in sterile Gifu anaerobic broth at 37°C until reaching the logarithmic phase under anaerobic conditions. The bacterial cells were harvested by centrifugation, washed, and resuspended in sterile 10 mM PBS (pH 7.2) to prepare a cell suspension with a concentration of 1 × 10^9^ cfu mL^−1^. For animal experiments, fresh bacterial cultures were prepared daily for gavage administration.

### Metagenomics analysis

The processing method for mouse fecal samples followed the same protocol as for 16S rRNA gene sequencing. Metagenomic sequencing was performed using the NovaSeq 6000 platform (Illumina, USA). Fastp software (version 0.20.0) was used to trim and remove low-quality reads, including reads with lengths less than 50 bp, average base quality below 20, or N bases. MEGAHIT software (version 1.1.2) was employed for high-quality reads assembly and merging, and contigs with lengths ≥300 bp were selected from the assembly results. MetaGene was used for open reading frames (ORFs) prediction of the contigs, and lengths ≥100 bp were translated into amino acid sequences based on the NCBI translation table (http://www.ncbi.nlm.nih.gov/Taxonomy/taxonomyhome.html/index.cgi?chapter=tgencodes#SG1). For gene sequence clustering, CD-HIT (version 4.6.1) was applied with 90% sequence identity and 90% coverage threshold, selecting the longest gene in each cluster as a representative sequence to build a non-redundant gene catalog. The SOAP aligner [23] software (version 2.21) was used to align high-quality reads from each sample to the non-redundant gene set (95% identity), enabling the computation of gene abundance information for each sample. The analysis of metagenomic results was conducted using the Majorbio cloud platform. The valid read number list for each sample is provided in supplementary Table S4.

### Statistical analysis

Statistical analyses were conducted using GraphPad Prism software (Version 6.0). Results are presented as mean ± standard deviation (SD). Statistical significance was assessed by one-way ANOVA followed by Tukey’s multiple comparisons, and *P* value of < 0.05 was considered a statistical difference. A Spearman’s correlation coefficient between samples greater than 0.6 or less than -0.6 and a *P* value less than 0.05 was considered statistically robust in correlation analysis. The α-Diversity index of community diversity (Shannon) and richness (Chao) were computed using Mothur v1.30.1 [24]. The similarity among microbial communities in different samples was assessed using principal coordinate analysis (PCoA) based on Bray-Curtis dissimilarity, employing the Vegan package (v2.5-3). Average linkage was employed for the hierarchical clustering method used in the analysis. Additionally, the amino acid sequences of the non-redundant gene set were compared to the NR database using BLASTP (with an e-value threshold of 1e-5) by Diamond [25] for taxonomic annotations. Functional category abundances were determined by aligning the sequences against the KEGG database (version 94.2).

## Results

### Preparation and characterizations of FOS and Levan

**Figure 1:**
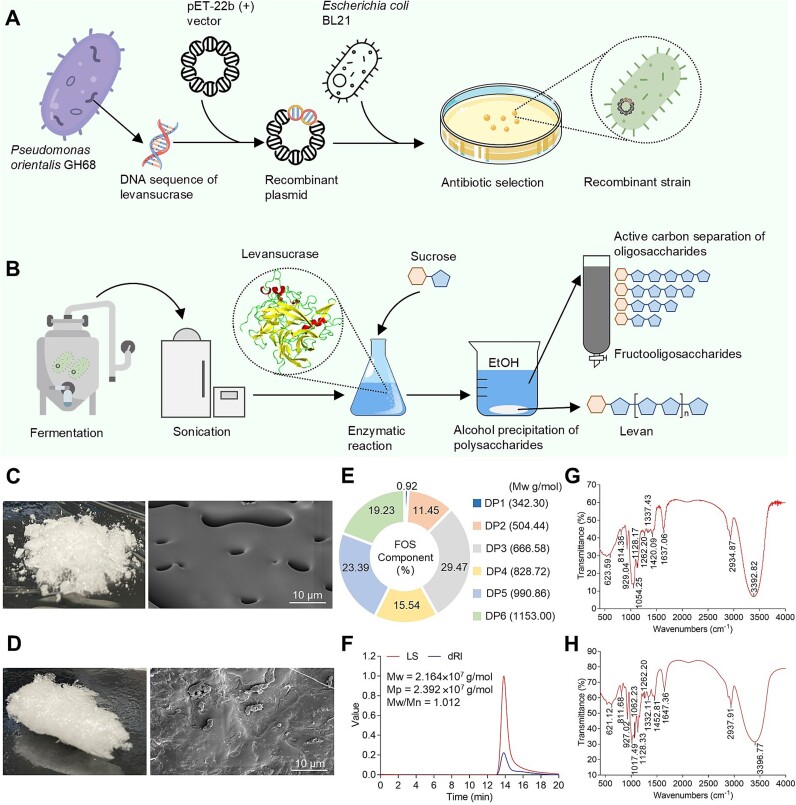
**Preparation and characterization of FOS and Levan.** (A) Construction process of *E. Coli* BL21 recombinants expressing levansucrase. (B) Preparation of levansucrase and the preparation and separation process of FOS and Levan. Characterization of FOS by scan electron microscope (C), ultra-performance liquid chromatography linear trap quadrupole orbitrap mass spectrometry (E), and fourier-transform infrared spectroscopy (G). Characterization of Levan by scan electron microscope (D), high-performance gel permeation chromatography-multi angle laser light scattering-refractive index (F), and fourier-transform infrared spectroscopy (H), where LS is light scattering detection, and dRI is differential refractive index.

Bioengineering provides the capability to customize and produce target products with high precision on a large scale [26]. To ensure that molecular weight is the only variable between FOS and levan, a combination of genetic engineering (Fig. 1A), fermentation engineering (Fig. 1B), and enzyme engineering was employed. Sucrose was used as the exclusive substrate, and the specificity of levansucrase (Supplementary Fig. S1) was leveraged to create levan-type fructans with different molecular weights. The obtained levan and FOS were subsequently purified separately through ethanol precipitation and activated carbon columns.

At both the macroscopic and microscopic level, FOS exhibited a porous and loose structure (Fig. 1C), whereas levan showed a dense structure (Fig. 1D). To determine the molecular weight and oligosaccharide composition of FOS, Ultra-performance liquid chromatography coupled with linear trap quadrupole orbitrap mass spectrometry was employed. The results indicated that the molecular weight of FOS ranges from 504 to 1153 Da. In contrast, the molecular weight of levan was analyzed using high-performance gel permeation chromatography combined with high-performance gel permeation chromatography-multi angle laser light scattering-refractive index (HPGPC-MALLS-RI). Average molecular weight (Mw) of levan was determined to be 2.164 × 10^7^ (±0.747%) Da (Fig. 1F). Additionally, the polydispersity index (Mw/Mn) of levan was found to be 1.012 (±1.049%), indicating a good uniformity. Purity assessments conducted via anthrone-sulfuric acid colourimetry revealed that the purity of FOS was 98.0%, while that of levan was 99.5%. Fourier-transform infrared (FT-IR) spectroscopy analysis revealed no significant differences in band intensities of functional groups between FOS and levan (Fig. 1G-H), indicating that the two obtained fructans were structurally similar but distinct in terms of molecular weight.

### Levan shows superior preventive efficacy than FOS in alleviating colitis

**Figure 2:**
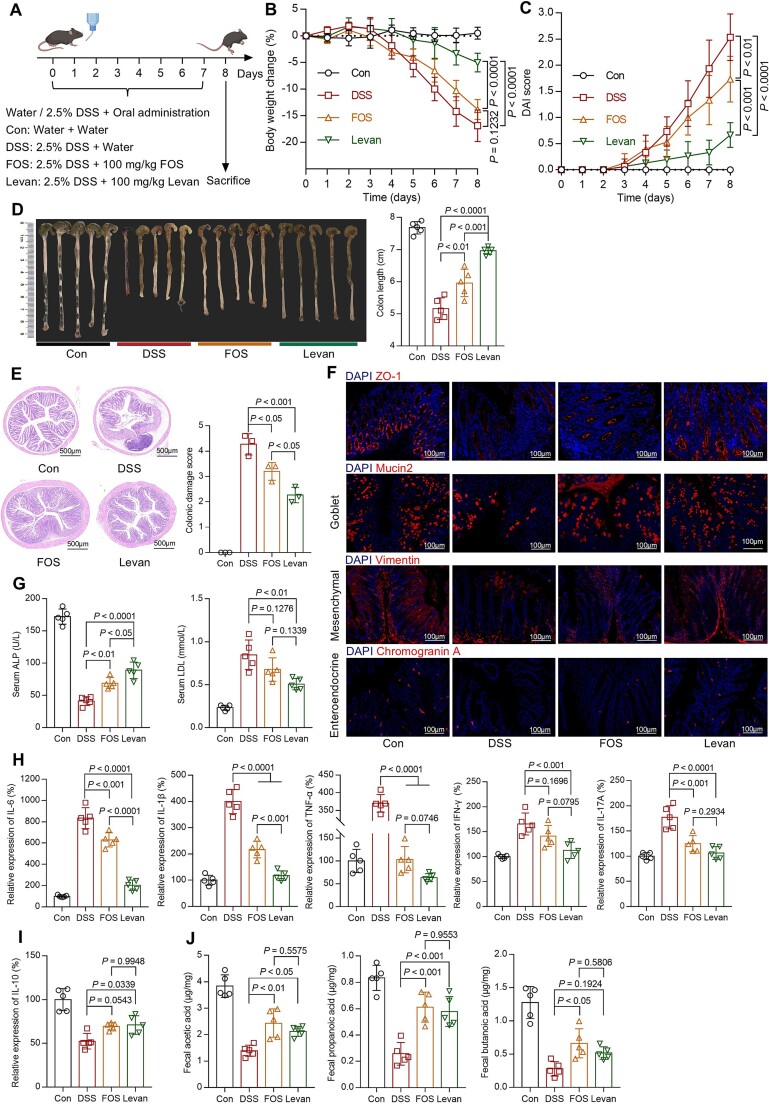
**Comparison of the preventive effects of FOS and Levan on colitis.** (A) C57BL/6 J mice were provided with water or 2.5% DSS-containing water for 7 d and orally administered with water or 100 mg kg^−1^ of FOS or Levan (*n* = 5). Body weight (B) and DAI score (C) were measured daily. On the 8^th^ day, after euthanizing the mice, quantitative measurements were taken for colon length (D), colon damage (E), expression of ZO-1, vimentin, mucin 2, and chromogranin a in colon tissue (F), serum inflammatory cytokines (G), pro-inflammatory (H) and anti-inflammatory (I) cytokine concentrations in the colon tissues, as well as SCFAs level in feces (J). The data are shown as the means ± standard deviation (SD) (n = 5). Statistical analysis was performed using one-way analysis of variance followed by Tukey’s multiple comparisons test.

To evaluate the differential therapeutic effects of fructans with varied Mw (FOS and levan) display, a mouse colitis model associated with gut dysbiosis was established using 2.5% DSS in drinking water for 7 days (Fig. 2A and Supplementary Fig. S3). Compared to the DSS group, both FOS and levan significantly alleviated DSS-induced colitis in mice (Fig. 2B-J). However, levan demonstrated superior preventive efficacy compared to FOS. Mice treated with levan exhibited a smaller decrease in body weight by 10% (Fig. 2B) and a significantly lower DAI score (*P <* 0.001, Fig. 2C). Moreover, the colon length in the levan group was more comparable to that of normal mice (Fig. 2D), demonstrating a superior protective effect on colonic epithelium (Fig. 2E). The cytosolic adapter zonula occludens-1 (ZO-1) is a crucial tight junction protein that bond adjacent cells in epithelial tissues [27]. Mesenchymal cells [28], goblet cells [29], and enteroendocrine cells [30] in the intestines play distinct roles in regulating intestinal structure, maintaining the integrity and homeostasis of the intestinal mucosa, and regulating metabolism and digestion, which are crucial for intestinal health. Immunofluorescence results showed that the quantity and status of ZO-1 and different cell types (mesenchymal cells, goblet cells, and enteroendocrine cells) of the levan group were closer to those of the normal group (Fig. 2F).

Certain protein biomarkers in serum reflect the overall health status of mice. Colitis is often accompanied by malnutrition, and low levels of ALP indicate a more severe state of malnutrition [31]. Following levan treatment, mice showed significantly higher ALP levels compared to the FOS group (*P <* 0.05, Fig. 2G). Elevated LDL levels promote inflammasome activation [32]. Levan treatment resulted in significantly lower LDL levels than the DSS group (*P <* 0.01, Fig. 2G), with no significant difference observed compared to the FOS group.

Treatment with either FOS or levan significantly reduced the levels of pro-inflammatory cytokines, such as IL-6, IL-1*β*, and TNF-*α*, in colonic tissues compared to the DSS group. Additionally, levan treatment exhibited significant inhibitory effects on pro-inflammatory cytokines INF-*γ* and IL-17A produced by T cells (Fig. 2H). However, short-term feeding of FOS and levan did not significantly increase the expression of the anti-inflammatory cytokine IL-10 (Fig. 2I). Therefore, compared to FOS, levan shows superior inhibition of pro-inflammatory cytokines in colonic tissue.

SCFAs are typically produced by gut microbiota through the fermentation of carbohydrates. To elucidate the differences in SCFAs production by gut microbiota due to FOS and levan, we quantified the levels of SCFAs using GC-MS analysis in mouse feces. The results revealed that FOS and levan treatment similarly resulted in higher levels of acetic acid and propionic acid in feces compared to the DSS group (Fig. 2J). FOS and levan led to a 2.3-fold (*P <* 0.05) and 1.85-fold (*P* = 0.1924) higher level of butyric acid, respectively. However, there were no significant differences in SCFA indices when comparing the FOS and levan groups.

### The colitis-alleviating effects of FOS and Levan are dependent on gut microbiota

**Figure 3:**
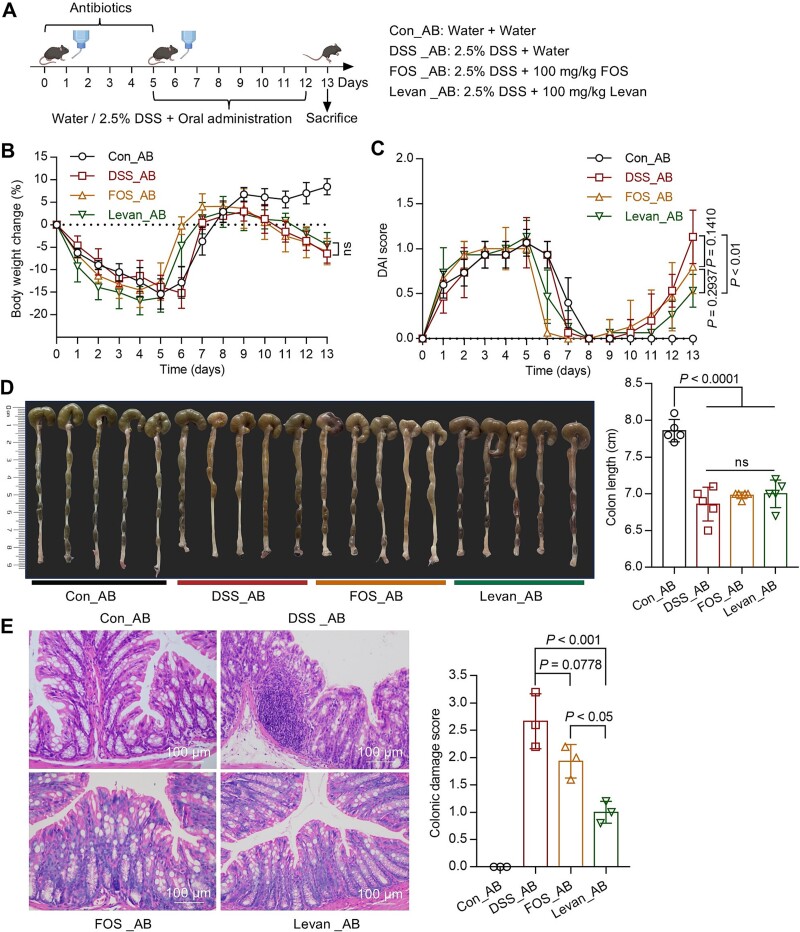
Depletion of gut microbiota abolished the preventive effects of FOS and Levan. (A) C57BL/6 J mice were pretreated for 5 days with broad-spectrum oral antibiotics added to the drinking water and then provided with water, or 2.5% DSS-containing water for 7 days, or with FOS and Levan treatments as described in the methods section (n = 5). Body weight (B) and DAI score (C) were measured daily. Colon length (D) and colon damage (E) were measured after euthanizing the mice. The data are shown as the means ± SD (n = 5). Each experiment was repeated twice. Statistical analysis was performed using one-way analysis of variance followed by Tukey's multiple comparisons test.

To determine whether the preventive effects of levan and FOS on colitis are dependent on gut microbiota modulation, mice were pretreated with a broad-spectrum oral antibiotics cocktail comprising ampicillin, vancomycin, neomycin, and metronidazole for a duration of 5 days to deprive gut microbiota (Fig. 3A and Supplementary Fig. S4). The results showed that the efficacy of FOS was completely abolished by antibiotics treatment (Fig. 3B-E). Conversely, while levan did not ameliorate the body weight loss and colon length in colitis mice pretreated with antibiotics, it significantly improved DAI score (*P <* 0.01, Fig. 3C) and showed restoration of the colonic epithelium (*P <* 0.001, Fig. 3E). Therefore, the efficacy of FOS is gut microbiota dependent, whereas the efficacy of levan is partially dependent on gut microbiota.

### Gut microbiota remodeling by Levan is effective and transmissible in action

**Figure 4:**
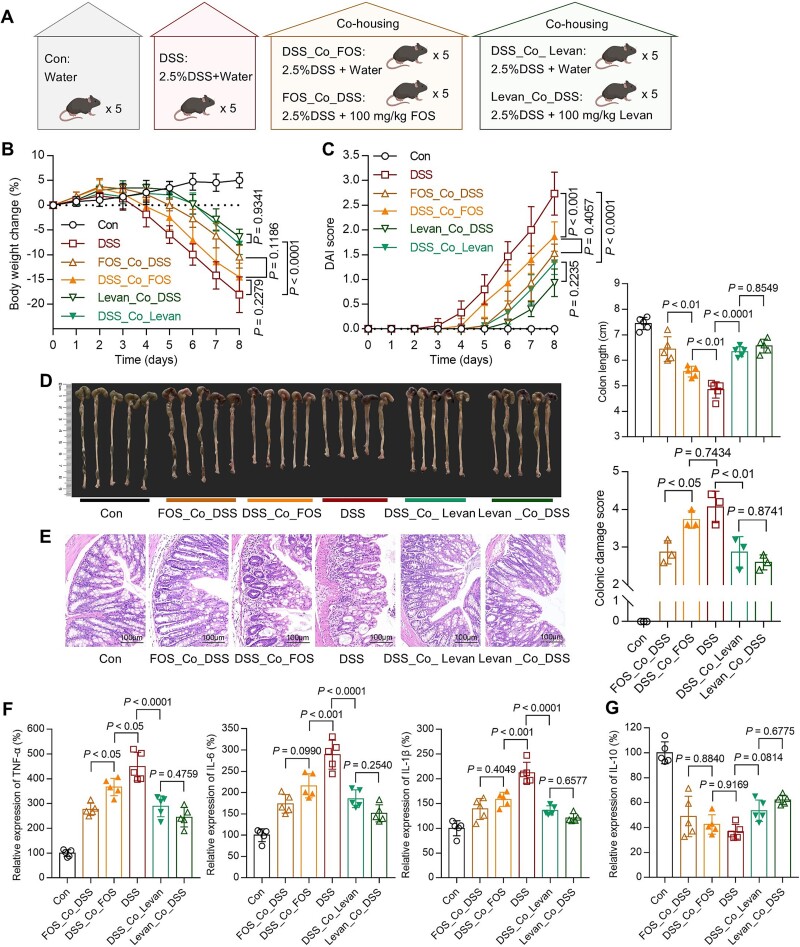
**Comparison of the preventive efficacy of co-housing on FOS and Levan.** (A) Ten C57BL/6 J mice were co-housed in the same cage and given the same 2.5% DSS and food. Five received daily oral administration of FOS or Levan, and the other five received water. Body weight (B) and DAI score (C) were measured daily. On the 8^th^ day, after euthanizing the mice, quantitative measurements were taken for colon length (D), colon damage (E), pro-inflammatory (F), and anti-inflammatory (G) cytokine concentrations in the colon tissues. The data are shown as the means ± SD (n = 5). Statistical analysis was performed using a one-way analysis of variance followed by Tukey’s multiple comparisons test.

Leveraging the coprophagic behavior of mice, which allows for the fecal-oral transfer of gut microbiota [33], we conducted co-housing experiments to further investigate the impact of these two dietary fructans on the gut microbiota. The design of the cohousing experiment is represented as shown in the figures (Fig. 4A, Supplementary Fig. S5A). Compared to the DSS group, mice of the DSS_Co_FOS group showed no significant differences in body weight loss (Fig. 4B) and colonic epithelial tissue damage (Fig. 4E). Furthermore, significant differences were observed as compared to the co-housed FOS-administered mice in terms of colon length (*P <* 0.01, Fig. 4D), colonic epithelial tissue damage (*P <* 0.01, Fig. 4E), and the levels of colonic pro-inflammatory cytokine TNF-*α* (*P <* 0.05, Fig. 4F). Conversely, mice of the DSS_Co_Levan group exhibited no significant differences as compared to the co-housed levan-treated mice, and showed alleviative body weight loss (*P <* 0.0001, Fig. 4B), decreased DAI score (*P <* 0.0001, Fig. 4C), improved colon length shortness (*P <* 0.0001, Fig. 4D), reduced colonic epithelial tissue damage (*P <* 0.01, Fig. 4E), and lower expression of colonic pro-inflammatory cytokines TNF-*α*, IL-6, and IL-1*β* (*P <* 0.0001, Fig. 4F), as compared to the DSS group mice. These findings revealed that both FOS and levan exhibited transferable effects on the gut microbiota of mice. In contrast, mice co-housed with orally administered levan showed superior efficacy and similarity. Additionally, we utilized high-performance liquid chromatography with charged aerosol detection to analyze the carbohydrate composition in the feces of mice from different groups. No levan signal was detected in the feces of the levan group (Supplementary Fig. S5B), indicating that the administered dosage of levan was completely metabolized. We excluded the possibility of levan molecules being present in the feces of mice treated with levan, suggesting that the impact of levan on gut microbiota was more effective and transmissible than that of FOS.

### Differential alterations of gut microbiota by FOS and Levan

**Figure 5:**
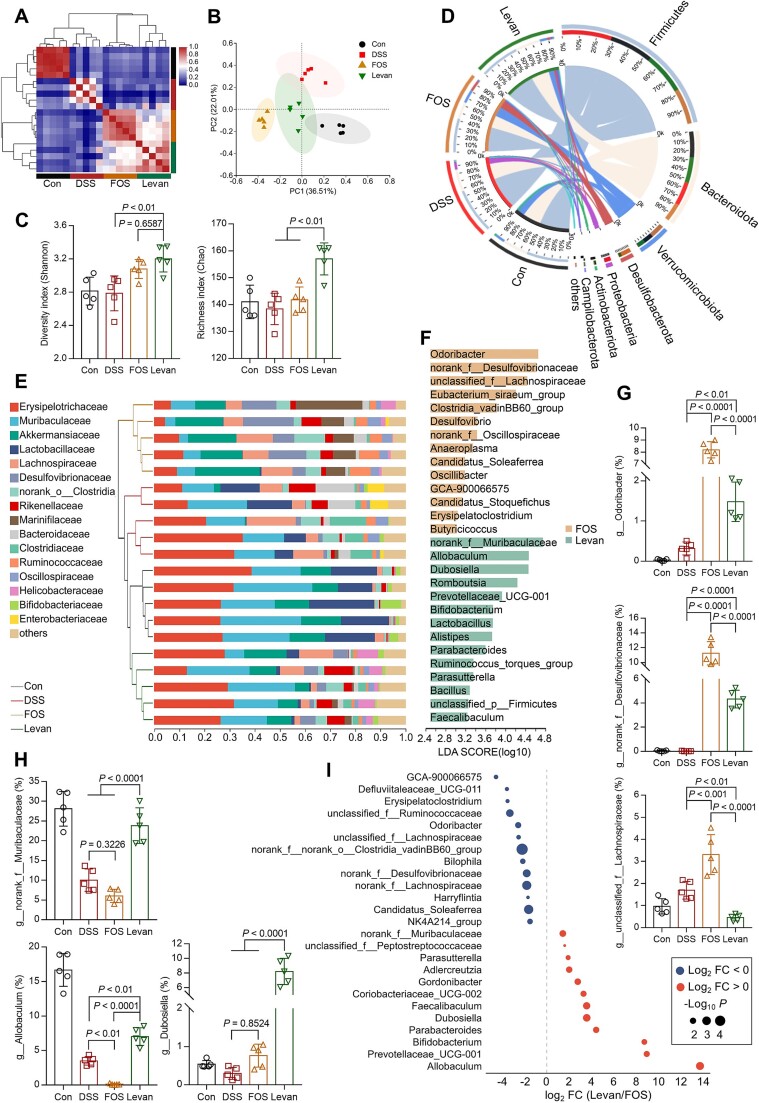
**Variations in gut microbiota composition across different treatment groups.** (A) Correlation analysis between samples at the OTU level. (B) PCoA of the gut microbiome composition on the OTU level among groups. The ovals represent the 95% confidence intervals of the species composition for each group of samples. (C) Analysis of *α*-diversity index of community diversity (Shannon) and richness (Chao). (D) Relative abundance of gut bacterial phylum among groups. (E) Relative abundance of gut bacterial family among samples. (F) LDA scores of differentially expressed genera between the FOS and Levan groups. The relative abundances of gut bacteria at the genus level were highly expressed in the FOS (G) or Levan (H) group. (I) Genera enrichment with |log_2_ FC (Levan/FOS)| > 1 in relative abundance. The data are shown as the means ± SD (n = 5). Statistical analysis was performed using one-way analysis of variance followed by Tukey’s multiple comparisons test.

Gut microbiota plays a significant role in the development and treatment of colitis [34]. Given that the protective effect of FOS and levan depended on gut microbiota, we examined the gut microbiota composition in colitis mice treated with FOS and levan. Analyses of 16S rRNA gene amplicon sequencing data revealed distinct gut microbiota profiles at the operational taxonomic unit (OTU) level among the four groups of mice (Fig. 5A-B). Based on the changes in gut microbiota composition, the samples in the DSS group exhibited low correlation (correlation value <0.3) with the samples from the FOS or levan group, indicating that FOS or levan treatment led to substantial microbial alterations in colitis mice. A higher similarity was observed between the FOS and levan groups (correlation value = 0.631, Supplementary Fig. S6). However, PCoA analysis further revealed that levan group samples were placed much closer to the control samples and were distinctly separated from the FOS group. This suggests that FOS and levan induced similar yet distinct microbial changes in colitis mice. Intervention by levan, but not FOS, led to a significantly higher bacterial diversity (Shannon) and richness (Chao) in mice compared to control and DSS groups. Although the Shannon indices were similar between the levan and FOS groups, levan treatment resulted in significantly higher richness in colitis mice compared to the FOS group (Fig. 5C). These results suggest that FOS and levan may exert differential prebiotic effects on gut microbiota.

Analysis at the phylum and family levels showed that levan treatment specifically resulted in a higher relative abundance of *Bacteroidota, Muribaculaceae,* and *Erysipelotrichaceae*, similar to the control group. In contrast, FOS primarily led to higher levels of *Desulfobacterota*, *Verrucomicrobiota,* and *Oscillospiraceae* (Fig. 5D-E). Linear discriminant analysis (LDA score > 3) effect size-based bar plots displayed the genera that best represented the largest differences between the FOS and levan groups (Fig. 5F). The bacterial genera enriched in feces from the FOS group (Fig. 5G) included *Odoribacter*, norank_f_*Desulfovibrionaceae*, and unclassified_f_*Lachnospiraceae*. Additionally, several genera from the *Oscillospiraceae* family, including *Eubacterium siraeum*, *Oscillibacter*, and others, were also observed (Supplementary Table S1). In contrast, characteristic genera *Turicibacter* (Supplementary Fig. S7), norank_f_*Muribaculaceae*, *Allobaculum,* and *Dubosiella* were more enriched in the levan group (Fig. 5H). Both *Allobaculum* and *Dubosiella* belong to the *Erysipelotrichaceae* family, which has been increasingly linked to inflammation [35] and metabolic disorders [36]. A Mendelian randomization study on gut metabolites documented that higher levels of the intestinal *Erysipelotrichaceae* family were causally related to a lower risk of inflammatory bowel disease (IBD) [37]. Furthermore, *Dubosiella* has been significantly enriched in treatments related to inflammation, especially in studies examining the effects of polysaccharides on the gut microbiota. In addition, genera with log_2_FC (levan / FOS) > 1, *P <* 0.05 in relative abundance were also displayed (Fig. 5I). The differential abundance of genera between the FOS and levan groups compared to the DSS group is presented (Supplementary Fig. S8).

### *D. Newyorkensis* exhibits preventive effects against colitis

**Figure 6:**
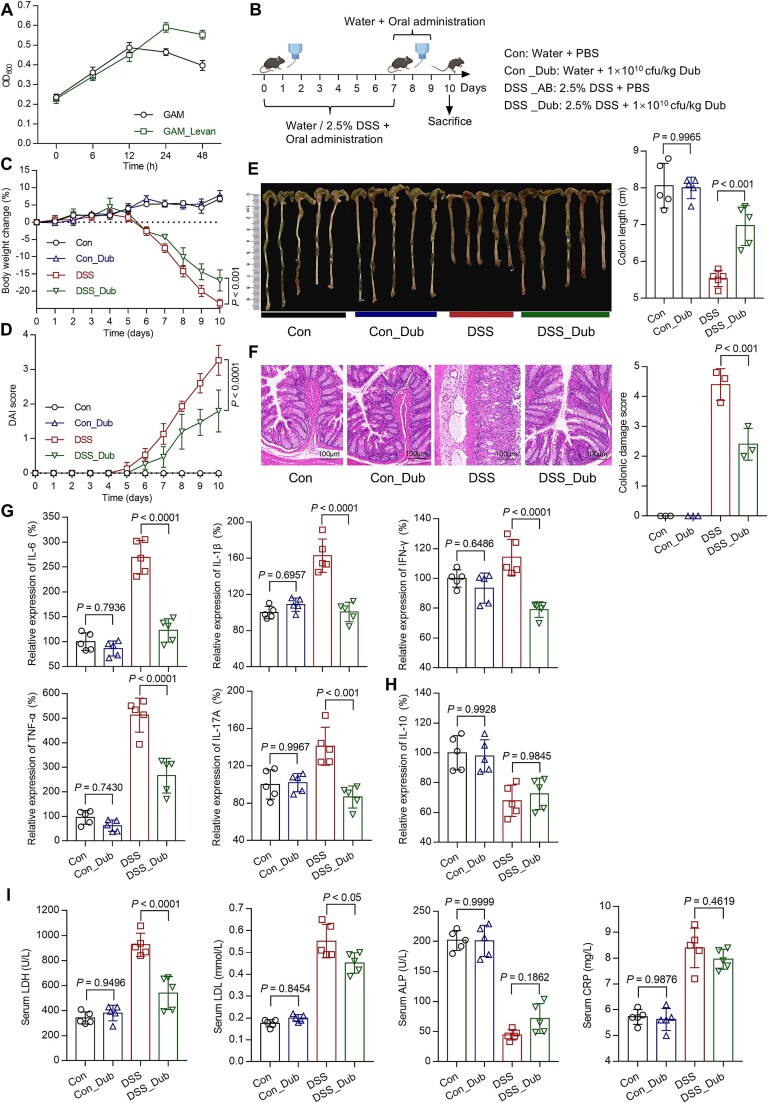
***D. Newyorkensis* exhibits preventive effects against colitis.** (A) In vitro culture of *D. Newyorkensis* with Levan. (B) C57BL/6 J mice were given water or 2.5% DSS-containing water for 7 days and orally administered with water or *D. Newyorkensis* (n = 5 for each group). Body weight (C) and DAI score (D) were measured daily. On the 10^th^ day, after euthanizing the mice, quantitative measurements were taken for colon length (E), colon damage (F), pro-inflammatory (G) and anti-inflammatory (H) cytokine concentrations in the colon tissues, as well as serum inflammatory cytokines. The data are shown as the means ± SD (n = 5). Statistical analysis was performed using one-way analysis of variance followed by Tukey’s multiple comparisons test.

To validate the impact of levan on *D. newyorkensis* and its functionality in colitis, the sole known strain of *Dubosiella*, *D. newyorkensis* NYU-BL-A4, was employed. *D. newyorkensis* is a strictly anaerobic, Gram-positive, non-spore-forming bacterium classified within the family *Erysipelotrichaceae*. Firstly, supplementation of levan in Gifu anaerobic medium (GAM) significantly promoted the growth of *D. newyorkensis* in vitro (Fig. 6A). Subsequently, to assess preventive potential of *D. newyorkensis* in DSS-induced colitis mice, mice were provided with drinking water containing or lacking 2.5% DSS for a continuous period of 7 days, during which they received oral gavage of water or *D. newyorkensis* for 10 days (Fig. 6B). The results showed that *D*. *newyorkensis* supplementation significantly reduced body weight loss (*P <* 0.001, Fig. 6C), DAI score (*P <* 0.01, Fig. 6D), colon length atrophy (*P <* 0.01, Fig. 6E), and demonstrated a significant protective effect on colonic epithelium (*P <* 0.001, Fig. 6F).

Further analysis showed that treatment with *D. newyorkensis* resulted in significantly lower levels of pro-inflammatory cytokines in colonic tissue compared to the DSS group, such as IL-6, IL-1*β*, TNF-*α*, INF-*γ*, and IL-17A (*P <* 0.001, Fig. 6G). Additionally, feeding of *D. newyorkensis* did not induce the expression of the anti-inflammatory cytokine IL-10 (Fig. 6H), in alignment with the effects observed following levan treatment. Analysis of inflammation-related factors in the serum revealed that supplementation with *D. newyorkensis* resulted in lower LDH (*P <* 0.0001) and LDL (*P <* 0.05) concentrations. However, no significant benefits were observed in ALP and CRP levels (Fig. 6I). Furthermore, no significant differences were noted in these indicators between normal mice supplemented with or without *D. newyorkensis*, indicating that oral administration of *D. newyorkensis* does not have adverse effects on normal mice.

### *D. Newyorkensis* selectively altered the composition of the gut microbiota

**Figure 7:**
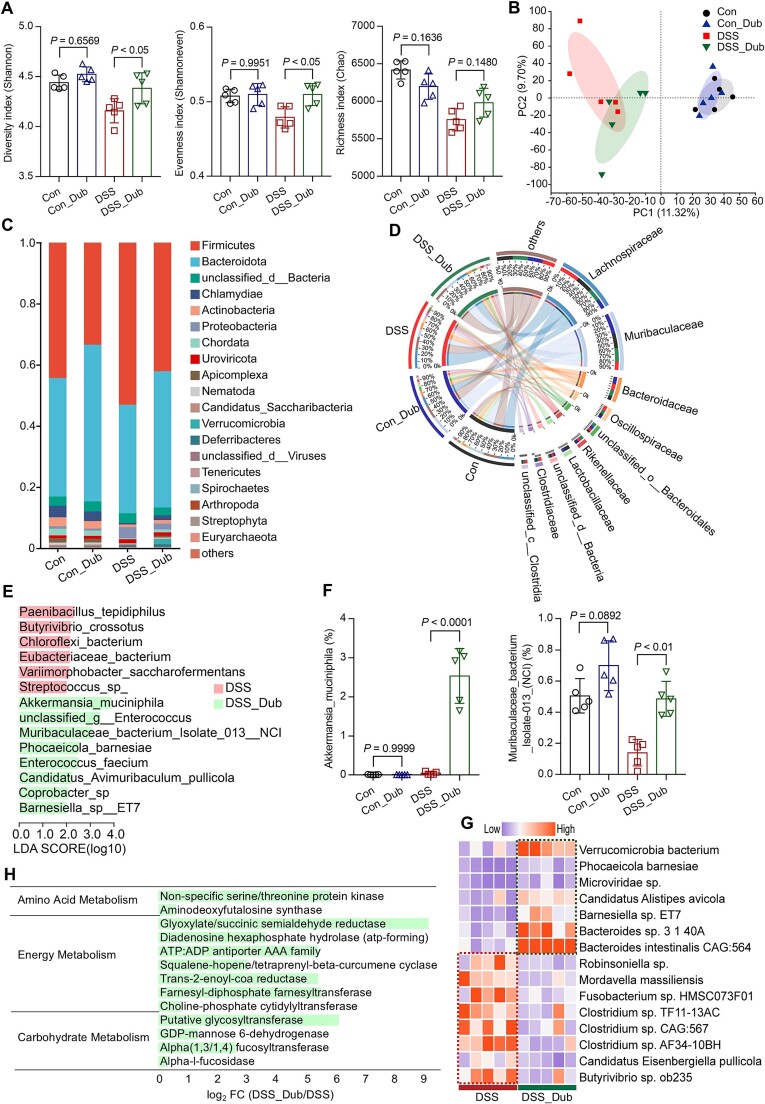
Modulation of gut microbiota by *D. Newyorkensis* supplementation. (A) Analysis of *α*-diversity index of community diversity (Shannon), evenness (Shannoneven), and richness (Chao). (B) PCA of the gut microbiome composition on the species level among groups. Relative abundance of gut bacterial phylum (C) and family (D) among groups. (E) LDA score of differentially expressed genera among group DSS and DSS_Dub. (F) Species with higher relative abundance after treatment with *D. Newyorkensis*. (G) the heatmap displays species with |log_2_FC (DSS_Dub/DSS)| > 1 and *P <* 0.05 in relative abundance. (H) Summary of significantly changed KEGG pathways based on metagenomic analysis of fecal samples from DSS_Dub and DSS group. Pathways with *P <* 0.05. The data are shown as the means ± SD (n = 5). Statistical analysis was performed using one-way analysis of variance followed by Tukey’s multiple comparisons test.

To further investigate the potential association between levan and the influence of *D. newyorkensis* on the intestinal microbiota, shotgun metagenomic data were acquired from mouse fecal samples with or without *D. newyorkensis* supplementation. We observed that supplementation with the single bacterial strain *D. newyorkensis* resulted in higher community diversity (Shannon) and evenness (Shannoneven) in colitis mice (Fig. 7A). The PCA at the species level also demonstrated distinct patterns between the DSS_Dub and DSS groups (Fig. 7B). Further analysis showed that supplementation with *D. newyorkensis* also led to a specific and significantly higher relative abundance of *Bacteroidota* (Fig. 7C), *Muribaculaceae* (Fig. 7D), and *Akkermansia muciniphila* [38–40] (Fig. 7E-F, known to degrade mucin). Additionally, *D. newyorkensis* inhibited the growth of *Clostridium* spp. (Fig. 7G). Comparison of the representative sequences from the non-redundant gene catalog with the KEGG database identified several affected pathways (Fig. 7H and Supplementary Fig. S10). The significant enrichment of pathways involved in amino acid metabolism, energy metabolism, and carbohydrate metabolism in the DSS_Dub microbiome suggests that *D. newyorkensis* and its associated bacterial network may enhance carbohydrate utilization, amino acid synthesis, and SCFA production.

## Discussion

For polysaccharides, the diversity of their types and functions can be attributed to variations in monosaccharide composition, the nature of glycosidic linkages, the degree of polymerization, and the extent of branching [41]. Although previous studies have shown that fructans exhibit modulatory effects on gut microbiota, a comparative analysis of the functional differences among different fructans has yet to be conducted. Our study elucidated the regulatory effects of FOS and levan on DSS-induced dysbiosis in mouse gut microbiota. Despite exhibiting significant differences in their degrees of polymerization, they share identical monosaccharide units and glycosidic linkages. Both FOS and levan interventions have been shown to ameliorate symptoms of colitis induced by DSS in mice (Fig. 2), including mitigating body weight loss, reducing colonic epithelial tissue damage, and decreasing local concentrations of pro-inflammatory cytokines in the colon. Additionally, levan demonstrated greater preventive efficacy in comparison to FOS. In contrast, FOS exhibited enhanced prebiotic characteristics as indicated by the levels of SCFAs. These findings suggest that differences in degrees of polymerization significantly influence the functionality of fructans, thereby affecting their prebiotic characteristics and their role in preventing the progression of colitis.

In mammals, the degradation of polysaccharides is primarily mediated by the gut microbiota [3]. Antibiotic experiments showed that the effectiveness of FOS is gut microbiota dependent (Fig. 3). Conversely, the effectiveness of levan is only partially dependent on the gut microbiota, as levan has been shown to confer protective effects on the colonic epithelial tissue of mice with compromised gut microbiota (Fig. 3F). This discrepancy may be ascribed to the higher degree of polymerization in levan compared to FOS, which leads to a more compact structure (Fig. 1C-D) and an increased solution viscosity (Supplementary Fig. S2B). Studies employing simulated digestion, in vitro fecal fermentation, and rheological models have revealed that fucoidan exhibited strong mucin adhesive properties within a simulated intestinal environment. This characteristic may improve the barrier properties of MUC and promote the mucosal delivery of functional components within the intestinal tract [42].

Various factors, including mucin and the turnover of gastrointestinal epithelial cells, limit the sustained delivery of drugs in the gastrointestinal tract [43]. Numerous drug delivery technologies designed for the gastrointestinal system are based on the principle of mucoadhesion, wherein the dosage form adheres to the mucosal surface to prolong contact time and improve absorption. For polysaccharides, higher molecular weight polymers typically require lower concentrations to achieve increased viscosity [44]. The interaction between dietary fibers and the mucus layer results in localized viscosity enhancements near the brush border in porcine models, thereby modulating the diffusion of nutrients across this barrier [45, 46].

16S rRNA gene sequencing was employed to evaluate alterations in the composition of the bacterial communities within the colonic lumen across various treatment groups. Overall, gut microbiota following treatment with FOS and levan exhibited a certain similarity (Fig. 5A). *A. muciniphila*, known for its immunomodulatory effects in the context of colitis [47], was significantly enriched in both groups (Supplementary Fig. S8). Through differential comparison, FOS mainly contributes to the enrichment of potent generators of SCFAs such as *Odoribacter* (known to produce SCFAs [48]), norank_f_*Desulfovibrionaceae* (known to produce acetic acid [49]), unclassified_f_*Lachnospiraceae* (known to produce butyric acid [50]) and several genera from the *Oscillospiraceae* family (known to produce butyric acid [51]). These enriched bacteria likely account for the elevated levels of SCFAs content in the feces of the FOS group when compared to the DSS group (Fig. 2J). In contrast, levan significantly resulted in higher richness of some bacterial groups compared to the FOS and DSS groups (Fig. 5C), primarily including *Turicibacter* (Supplementary Fig. S7), known to modify bile acids and host lipids [52], norank_f_*Muribaculaceae* (known to reduce *Clostridiodes difficile* colonization [53]), *Allobaculum* (known to possess immunomodulatory properties [54, 55]). This indicates that levan’s influence on the gut microbiota is reflected not only in the production of SCFAs but also in reducing the colonization of pathogenic bacteria and exerting an immunomodulatory effect in the intestine. Polysaccharides have also been shown to modulate gut microbiota and immune cell function in numerous studies [56–58]. Co-housing experiments clarified the transmissibility of gut microbiota among hosts (Fig. 4) and further demonstrated that the regulatory and beneficial effects of levan on the gut microbiota can be stably transmitted via the microbiota. Other bacteria negatively correlated with intestinal diseases were also enriched in the levan group, including *Romboutsia* [59, 60], *Lactobacillus* [61, 62]*, Prevotellaceae UCG-001* [63–66], *Alistipes* [67–69], and *Parabacteroides* [13, 70, 71].

Studies focused on the prebiotic effect of polysaccharides on gut microbiota have documented that *Dubosiella* abundance was higher in treatment groups compared to disease groups through 16S rRNA gene sequencing [72, 73]. Furthermore, *Dubosiella* was found to be specifically enriched in the levan treatment group (Fig. 5F). In contrast, the abundance of *Dubosiella* in the FOS group showed no significant difference compared to the normal and DSS groups. This may indicate that *Dubosiella* exhibits a preference for high molecular weight fructans. Presently, the only identified strain within the genus *Dubosiella* is *D. newyorkensis*, which has been demonstrated to restore the balance of Treg/Th17 responses and mitigate mucosal barrier damage through the production of propionate and L-Lysine from its live bacteria or culture supernatant [74]. In our analysis of differential KEGG pathways based on the metagenomic of fecal samples from the DSS_Dub and DSS groups (Fig. 7H), we identified two pathways significantly upregulated in the DSS_Dub microbiome, non-specific serine/threonine protein kinase [EC:2.7.11.1] and aminodeoxyfutalosine synthase [EC:2.5.1.120]. These pathways regulate key enzymes in L-lysine production through protein phosphorylation and menaquinone biosynthesis [75]. Glyoxylate/succinic semialdehyde reductase [EC:1.1.1.79] with a Log_2_ FC (DSS_Dub/DSS) above 9 supports propionate synthesis by influencing succinate metabolism [76]. Additionally, trans-2-enoyl-CoA reductase [EC:1.3.1.44] facilitates this process by modulating fatty acid *β*-oxidation, a pathway that can lead to the production of SCFAs [77]. Furthermore, we identified 8 unique carbohydrate-active enzyme families that were exclusively present in both the Con_Dub and DSS_Dub groups (Supplementary Fig. S11), primarily including glycoside hydrolase (GH5_11, GH5_18, GH5_20, and GH5_15) and glycosyltransferase family (GT16 and GT38). These results suggested that *D. newyorkensis* and its affected bacterial network have a preference for polysaccharide metabolism.

Further functional experiments were carried out on *D. newyorkensis* and confirmed its significant preventive effect on colitis (Fig. 6). Additionally, the metagenomic analysis revealed that the changes in gut microbiota induced by *D. newyorkensis* were characterized by promoting the proliferation of *Bacteroides* and *Muribaculaceae*. The administration of *D. newyorkensis* was found to promote the proliferation of *A. muciniphila* in the gut microbiota under colitis conditions (Fig. 7F), a bacterium recognized for its protective role in colitis [78]. This finding suggests that *D. newyorkensis* can restrict DSS-induced colitis by modulating the gut microbiota.

## Conclusion

The findings of this study reveal the distinct modulatory effects of fructans with varying molecular weights on gut microbiota. Specifically, low molecular weight FOS primarily function as a generator of SCFAs. In contrast, high molecular weight levan not only enhances SCFA production but also contributes to higher diversity and richness of the gut microbiota, thereby offering additional benefits. In particular, *Dubosiella*, which was enriched following levan treatment, has the potential to serve as a probiotic for the prevention and treatment of colitis, as well as other gastrointestinal disorders such as colon polyps and colon cancer. Furthermore, the exploration of bioactive compounds in *Dubosiella* cultures or fecal samples from mice post-treatment, facilitated by advancements in metabolomics, could enhance our understanding of the complex interactions between probiotics and prebiotics, as well as their implications for disease management, particularly regarding the relationship between fructans and *Dubosiella* in this investigation.

## Supplementary Material

Supplementary_Information_wrae262

Taxonomy_Profile_of_16S_rRNA_and_Metagenomics_wrae262

## Data Availability

Raw data from 16S rRNA gene sequencing and metagenomics have been presented in the National Center for Biotechnology Information (NCBI) Sequence Read Archive (SRA) database under accession numbers PRJNA1051022 and PRJNA1051679. SCFAs quantification raw data has been deposited in Mendeley Data (https://data.mendeley.com/datasets/f84ms3gm25/1).
