# Supplementary material for: Polymerization of dietary fructans differentially affects interactions among intestinal microbiota of colitis mice
Source: ISME J. 2025 Jan 2;19(1):wrae262. doi: 10.1093/ismejo/wrae262 (PMC11742283; doi:10.1093/ismejo/wrae262)
Supplement: Supplementary_Information_wrae262 [file supplementary_information_wrae262.pdf]

Supplementary Information for

Polymerization of dietary fructans differentially affects interactions among intestinal microbiota of colitis mice

Yaqin Xiao, Qianyun Zhao, Dawei Ni, Xiaoqi Zhang, Wei Hao, Qin Yuan, Wei Xu, Wanmeng Mu, Dingtao Wu, Xu Wu, Shengpeng Wang

Supplementary Methods	2
Supplementary Figure 1	7
Supplementary Figure 2	8
Supplementary Figure 3	9
Supplementary Figure 4	10
Supplementary Figure 5	11
Supplementary Figure 6	12
Supplementary Figure 7	13
Supplementary Figure 8	14
Supplementary Figure 9	15
Supplementary Figure 10	16
Supplementary Table 1	17
Supplementary Table 2	18
Supplementary Table 3	19
Supplementary Table 3	20

Supplementary materials and methods

Levansucrase purification

E. coli pellets were dissolved in lysis buffer (50 mM PBS pH 7.0, 100 mM NaCl) and solubilized by sonication. After centrifugation (8000 *g*, 30 min, 4°C), the supernatant was applied (5 mL min^{-1}) to a 5 ml HisTrap HP column (Cat. #17524701, Cytiva) with an AKTA Pure system (GE Healthcare), previously equilibrated with binding buffer (50 mM PBS pH 7.0, 500 mM NaCl). After washing the column with binding buffer for 5 min, it was further washed with washing buffer (50 mM PBS pH 7.0, 500 mM NaCl, 50 mM imidazole) until the absorbance signal (280 nm) returned to the baseline. The protein of interest was eluted by a linear gradient 10-100% elution buffer (50 mM PBS pH 7.0, 500 mM NaCl, 500 mM imidazole) until the absorbance signal (280 nm) no longer decreased. Fractions containing sufficient concentrations of pure protein were pooled and dialyzed for 4 hours at 4°C against 1 L of dialysis buffer (50 mM PBS pH 7.0, 10 mM EDTA-2Na). Subsequently, the sample was further dialyzed overnight at 4°C using 50 mM PBS (pH 7.0). The molecular mass and purity of the purified enzyme was checked by sodium dodecyl sulfate polyacrylamide gel electrophoresis (Cat. #PG113, Yamei) and the concentration was measured by using a Nanodrop 2000C spectrophotometer (Thermo Scientific) at 280 nm.

Levan and FOS Production

The reaction was carried out in a 100 mL system with 50 mM PBS (pH 6.5), 20 mg L^{-1} purified enzyme, and 300 g L^{-1} sucrose. The reaction was performed at 45°C for 6 h and terminated by boiling in water bath for 10 min. After centrifuging at 8000 rpm for 10 min, 95% ethanol was slowly added dropwise to the supernatant until the mixture concentration of ethanol reached 65%, stood overnight at 4°C and subsequently centrifuged at 8000 rpm for 10 min to collect the precipitate. The supernatant was retained for the preparation of FOS. The precipitate was washed three times until the supernatant showed no monosaccharide detection using High-Performance Liquid Chromatography (HPLC, Agilent 1200 system, Agilent technologies). After vacuum

freeze-drying, pure levan was obtained.

After removing ethanol from the supernatant containing FOS using rotary evaporation at 55°C, active carbon adsorption chromatography was used to remove monosaccharides and sucrose. Elution was performed at 5%, 10%, 15%, 20%, 30% and 95% ethanol at a flow rate of 1 mL min^{-1}. One tube was collected every 10 mL, and the content of FOS was measured using HPLC. Fractions containing sufficient concentrations of FOS were pooled and lyophilized.

The total carbohydrate content was determined using the phenol-sulfuric acid method. The test solution was diluted to a concentration of 0.05 mg mL^{-1}. From this, 400 μL of the sample was taken, and 200 μL of 6% phenol was added and mixed thoroughly. Subsequently, 1 mL of sulfuric acid was added to the mixture. The solution was then heated in a water bath at 90°C for 10 minutes, immediately cooled, and the absorbance was measured at 490 nm. The carbohydrate content was calculated using a standard curve based on the absorbance values (Supplementary Fig. S2A).

Molecular weight determination

The molecular weight of levan was measured using high-performance gel permeation chromatography-multi angle laser light scattering-refractive index (HPGPC-MALLS-RI) and performed on Shimadzu HPLC System and DynaPro NanoStar Dynamic Light Scattering Detector (Wyatt Technology Co.) equipped with BRT105-104-102 tandem gel column (8 × 300 mm, BoRui Saccharide Biotech Co., Ltd). The sample was eluted with a 50 mM NaCl solution at 25°C with a flow rate of 1 mL min^{-1}. The dn/dc value was 0.1380 mg L^{-1} at 658.1 nm. Data was analyzed using Astra software version 8.

Quantitative analysis of the components of FOS was performed using the ultra-performance liquid chromatography linear trap quadrupole orbitrap mass spectrometry platform. 10 mg of FOS is mixed with 1.5 mL of methanol and 3 mL of dichloromethane, and incubated at room temperature for 1 hour. Add 1.25 mL of water and let it sit at room temperature for 10 minutes. Evaporate to dryness and add 200 μL of a mixture

(dichloromethane: methanol: water = 60: 30: 4.5). Take 250 μL of the sample and add 750 μL of acetonitrile, centrifuge at 13,000 r min^{-1} for 10 minutes at 4°C. Collect the supernatant for LC-MS analysis. Chromatographic conditions: Waters UPLC BEH C18 column (100 mm × 2.1 mm, 1.7 μm); mobile phase: 0.1% formic acid in water (A) and 0.1% formic acid in acetonitrile (B); flow rate: 0.3 mL min^{-1}; column temperature: 40°C; injection volume: 5 μL; data collection in both positive and negative ion modes. Use Progenesis QI software (Nonlinear Dynamics, Durham, NC, USA) to align peaks and extract peaks from RAW data files.

Scan electron microscope (SEM) inspection

5 mg of the dried FOS and levan were adhered to the conductive carbon film coated with double-sided adhesive, further placed in the ion sputtering chamber for gold sputtering for ca. 40 seconds. Afterward, the sample was subjected to the SEM (Nova NanoSEM 450, Field Electron and Ion Company) observation chamber, where their surfaces were inspected at an accelerating voltage of 5 kV.

Fourier-transform infrared (FT-IR) analysis

FOS and levan samples were dried in a 50°C oven and then mixed separately with solid KBr at a ratio of 1:100. The mixture was ground and then compressed into a pellet. The spectrum of the pellet was recorded amid 4000-400 cm^{-1} versus neat KBr pellet (blank) and analyzed using FT-IR spectrometer (Nicolet iS10, Thermo Scientific).

FOS and levan treatment

After acclimation, the DSS (n = 5), FOS (n = 5), and Levan groups (n = 5) were given 2.5% DSS in drinking water for 7 days and were respectively treated daily with water, 100 mg kg^{-1} FOS, and 100 mg kg^{-1} Levan by oral gavage. The Con group (n = 5) received normal water and was administered 0.2 mL of water by oral gavage daily. Mice were sacrificed on the 8th day, following the method described in section 2.2.

Polysaccharide detection in fecal samples

A frozen fecal sample of 100 mg was ground, and 1 mL of water was added. The mixture was homogenized and subjected to ultrasonic extraction. The sample was

centrifuged at 8000 r min^{-1}, and the supernatant was collected. The supernatant was filtered through a 0.45 μm aqueous phase membrane. High-performance liquid chromatography with a charged aerosol detector (HPLC-CAD) was used for detection, with 0.02 M ammonium acetate as the mobile phase. The separation was performed using an Acclaim SEC-1000 gel column (7 μm, 7.8 x 300 mm) at 40°C, with a flow rate of 0.6 mL min^{-1}.

Antibiotic treatment

For *in vivo* antibiotic treatment, mice were treated with a broad-spectrum oral antibiotics cocktail including 1 g L^{-1} ampicillin (Goldbio, USA), 1 g L^{-1} metronidazole (M) (Sigma), 1 g L^{-1} neomycin (Sigma), and 0.5 g L^{-1} vancomycin (V) (Sigma) in sterile distilled water for 5 days. On the 5th day, after discontinuing the combined antibiotics, the DSS (n = 5), FOS (n = 5), and Levan groups (n = 5) were given 2.5% DSS in drinking water for 7 days and were respectively treated daily with water, 100 mg/kg^{-1} FOS, and 100 mg kg^{-1} Levan by oral gavage. The Con group (n = 5) received normal water and was administered 0.2 mL of water by oral gavage daily. Mice were sacrificed on the 8th day, following the method described in section 2.2.

Co-housing experiment

The Con group (n = 5) was given normal water and administered 0.2 mL of water by gavage daily. Other groups received 2.5% DSS in drinking water for 7 days. The DSS group (n = 5) was treated with 0.2 mL of water by gavage daily (n = 5). In the co-housing cage, daily gavage of 100 mg/kg FOS to mice numbered 1-5 was designated the FOS_Co_DSS group, while gavage of water to mice numbered 6-10 was designated the DSS_Co_FOS group. The same procedure was applied to levan. Mice were sacrificed on the 8th day, following the method described in section 2.2.

***D. newyorkensis* treatment**

The Con (n = 5) and Con_Dub groups (n = 5) were given normal drinking water and treated with 0.2 mL 10 mM PBS (pH 7.2) or 1×10^{10} CFU kg^{-1} *D. newyorkensis* by gavage (n = 5) for 10 days. The DSS (n = 5) and DSS_Dub groups (n = 5) received 2.5%

DSS in drinking water for 7 days, followed by normal drinking water for the next 3 days, and were treated with 0.2 mL 10 mM PBS (pH 7.2) or 1×10^{10} CFU kg^{-1} *D. newyorkensis* by gavage (n = 5) for 10 days. Mice were sacrificed on the 10th day, following the method described in section 2.2. *D. newyorkensis* were cultured daily for gavage administration to ensure viability.

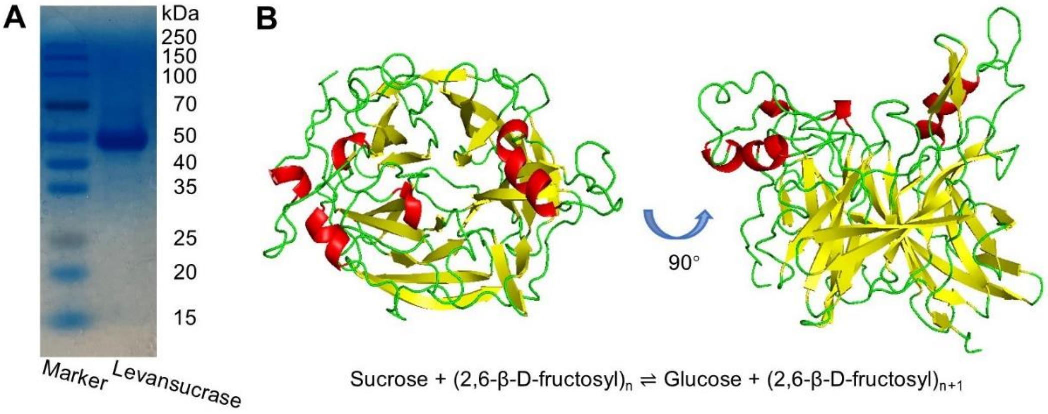

Supplementary Figure 1. **The molecular weight and structure of levansucrase.** Related to Figure 1B. SDS-PAGE analysis (**A**) and 3D structure (**B**) of levansucrase from *Pseudomonas orientalis* GH68.

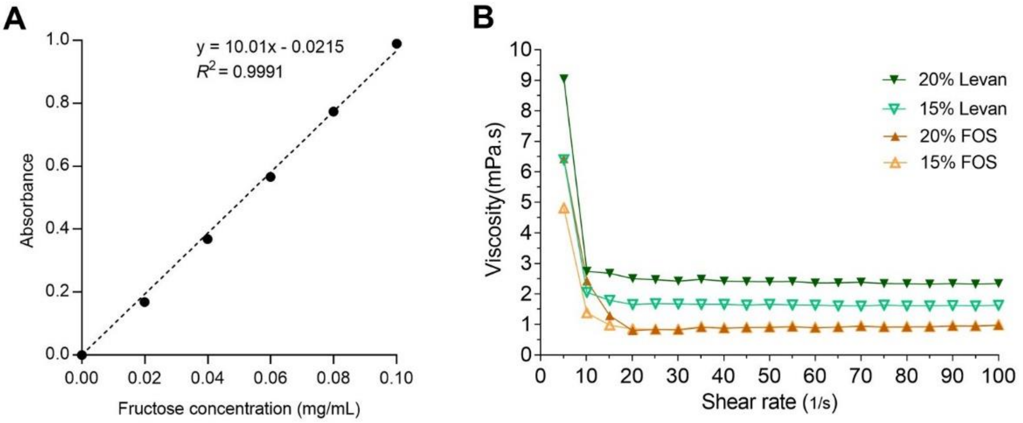

Supplementary Figure 2. (**A**) The standard curve of different concentrations of fructose using anthrone-sulfuric acid colorimetry. (**B**) Flow curves of different FOS and levan concentrations.

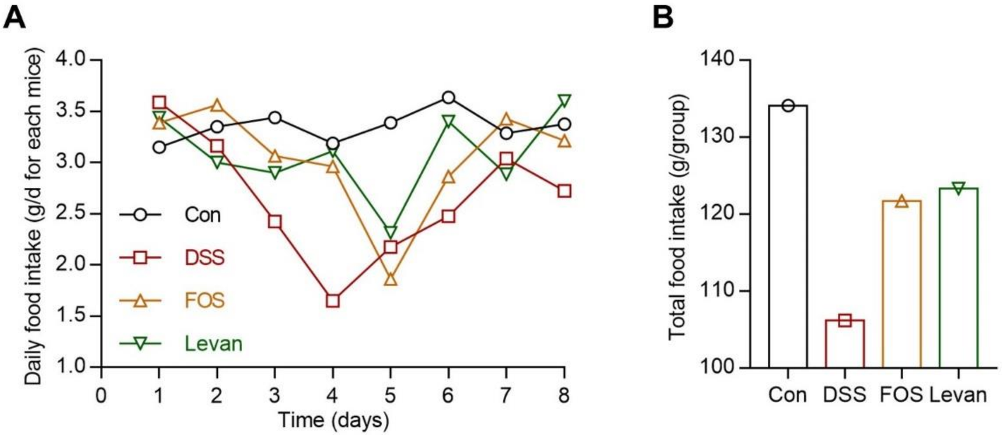

Supplementary Figure 3. Comparison of dietary changes in colitis mice with FOS and levan. (A) Average daily food intake per mice in each group. (B) Total food intake over 8 days for each group of mice during the experimental period. Related to Figure 2.

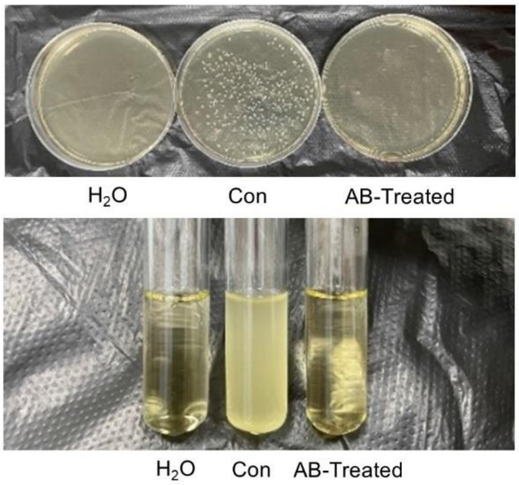

Supplementary Figure 4. Fecal cultures of mice on day 5 in antibiotic experiment. Related to Figure 3. H_2O group was cultured in LB solid or liquid medium without feces for 24 hours. Con group contained a dilution of fecal suspension from normal mice. AB-Treated group contained a dilution of fecal suspension from mice that had been treated with antibiotics for 5 days.

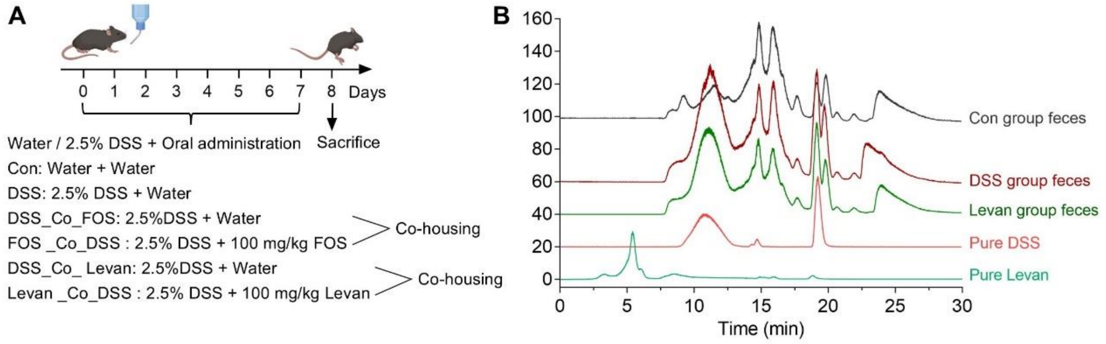

Supplementary Figure 5. (A) Schematic Diagram of the Co-housing Experiment. C57BL/6J mice were given water or 2.5% DSS-containing water for 7 d and orally administered with water or 100 mg kg^{-1} of FOS or levan (n = 5 for each group). Related to Figure 4A. (B). Detection of carbohydrates in feces of different mouse groups using high-performance liquid chromatography with charged aerosol detection

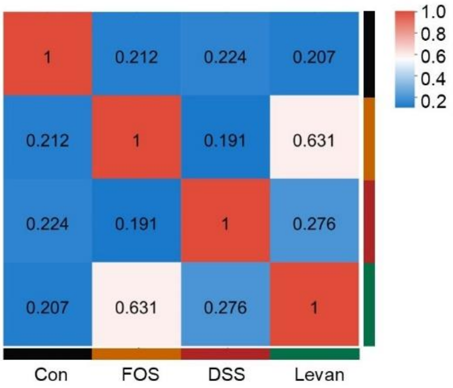

Supplementary Figure 6. **Correlation analysis between groups at the OTU level.** Related to Figure 5A. Samples in four groups were analyzed using 16S rRNA gene amplicon sequencing. The data are shown as the means ± SD (n=5).

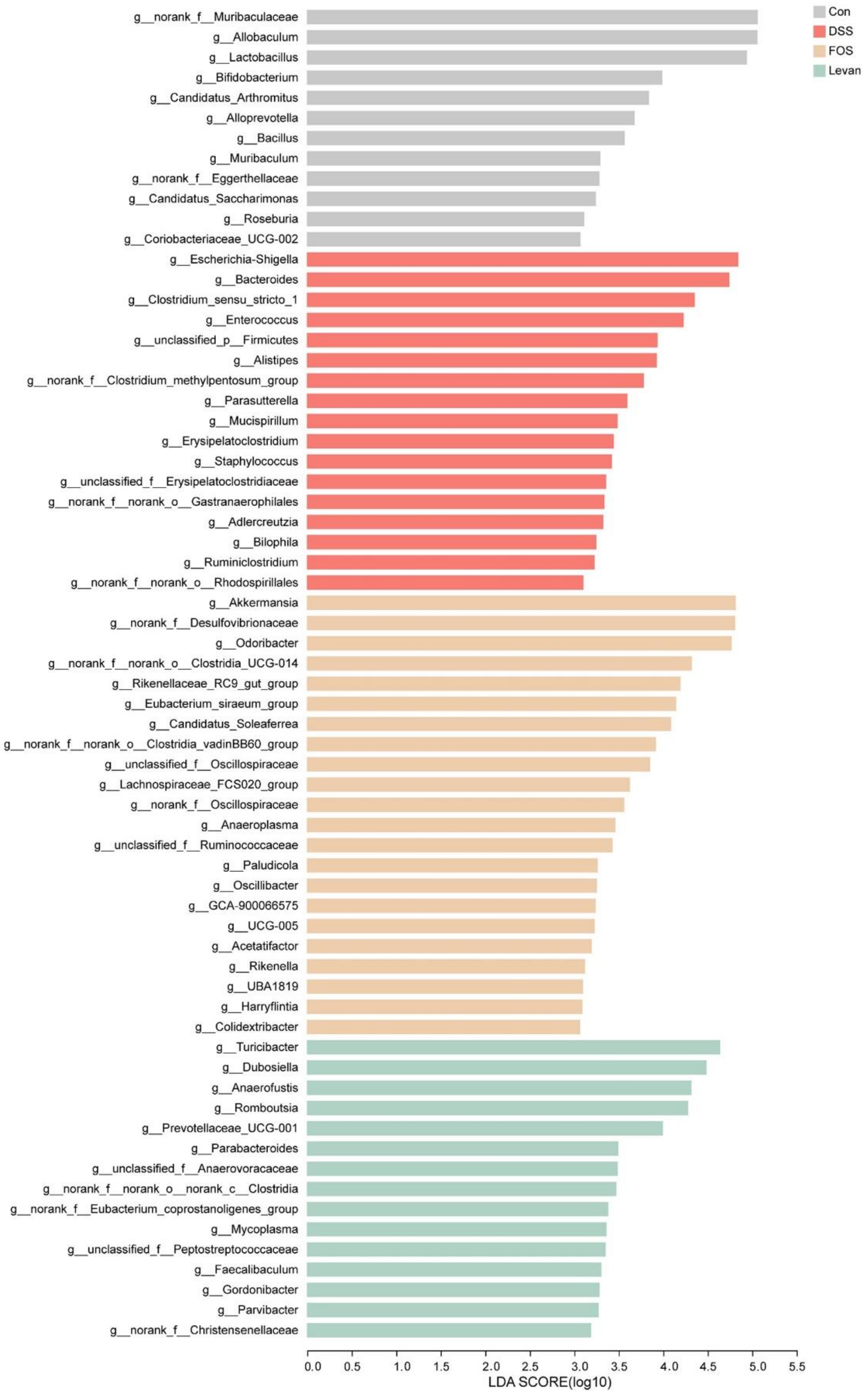

Supplementary Figure 7. **Linear discriminant analysis effect size-based bar plot expressed differentially abundant genera among groups.** Related to Figure 5F. Samples in four groups were analyzed using 16S rRNA gene amplicon sequencing. The data are shown as the means ± SD (n=5).

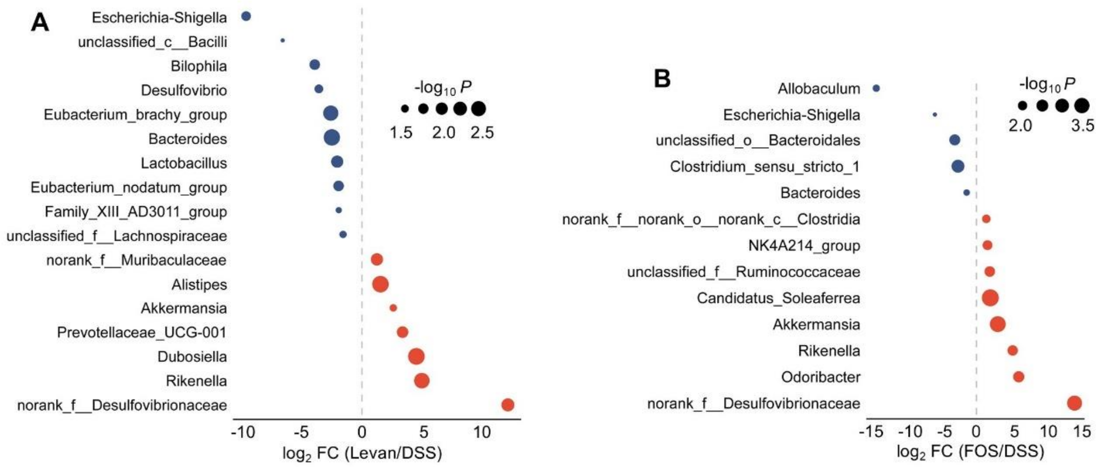

Supplementary Figure 8. Genera enrichment with |log$_2$ FC (Levan/DSS)| > 1 (**A**) and |log$_2$ FC (FOS/DSS)| > 1 (**B**) in relative abundance ($P < 0.05$). Related to Figure 5I. Samples in three groups were analyzed using 16S rRNA gene amplicon sequencing. The data are shown as the means ± SD (n=5).

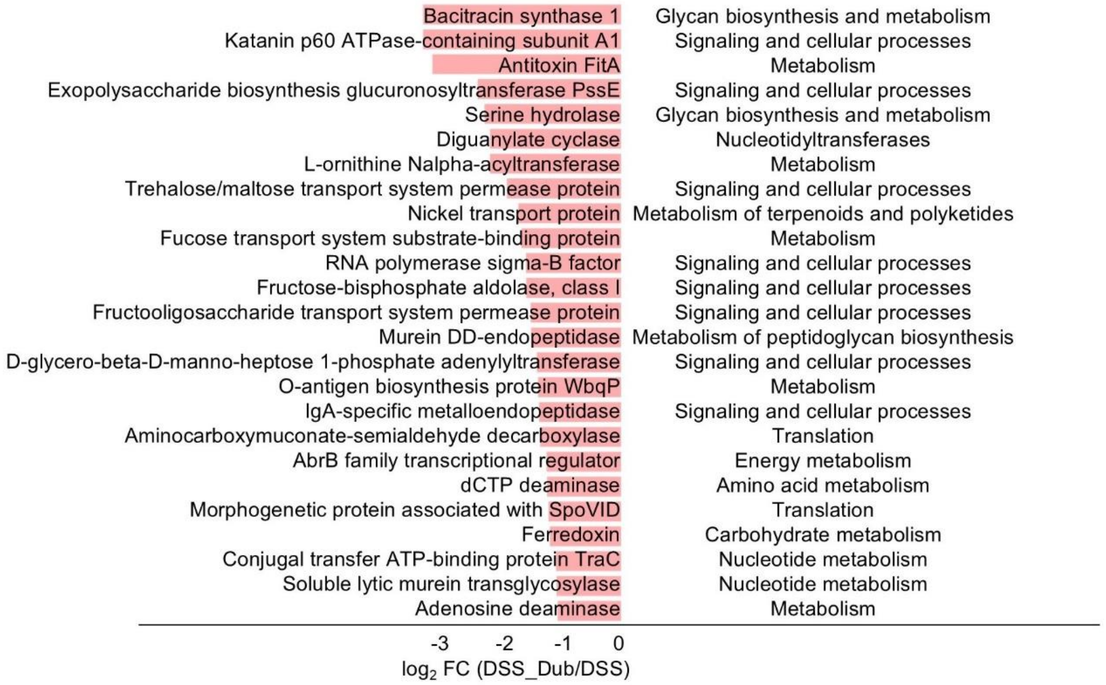

Supplementary Figure 9. Summary of significantly changed KEGG pathways based on matagenomic analysis of fecal sample from DSS_Dub and DSS group. Pathways with $\log_2$ FC (DSS_Dub/DSS) < 1 and $P < 0.05$. Related to Figure 7H. The data are shown as the means ± SD (n=5).

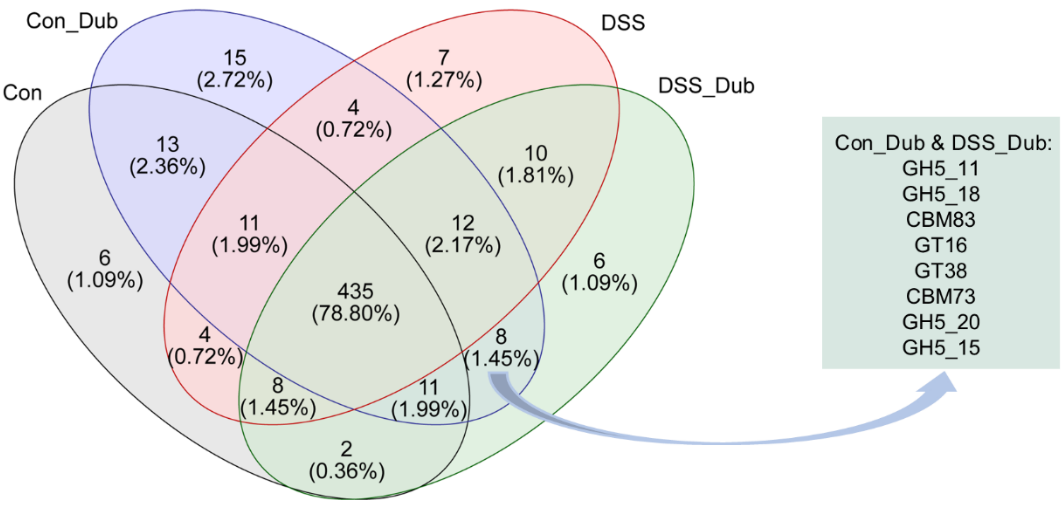

Supplementary Figure 10. The venn diagram reveals overlaps and uniqueness of carbohydrate-active enzymes in groups. Related to Figure 7. Samples in four groups were analyzed using shotgun metagenomic sequencing. The data are shown as the means ± SD (n=5).

Supplementary Table 1. Linear discriminant analysis score and taxonomic classification of bacterial gene among group FOS and levan. Related to Figure 5F.

Group	LDA Score	Phylum	Class	Order	Family	Genus
FOS	4.65	Bacteroidota	Bacteroidia	Bacteroidales	Odoribacteraceae	Odoribacter
FOS	4.61	Thermodesulfobacteriota	Desulfovibrionia	Desulfovibrionales	Desulfovibrionaceae	norank_f__Desulfovibrionaceae
FOS	4.43	Firmicutes	Clostridia	Eubacteriales	Lachnospiraceae	unclassified_f__Lachnospiraceae
FOS	4.20	Firmicutes	Clostridia	Eubacteriales	Oscillospiraceae	Eubacterium_siraeum_group
FOS	3.82	Firmicutes	Clostridia	norank	norank	Clostridia_vadinBB60_group
FOS	3.43	Thermodesulfobacteriota	Desulfovibrionia	Desulfovibrionales	Desulfovibrionaceae	Desulfovibrio
FOS	3.42	Firmicutes	Clostridia	Eubacteriales	Oscillospiraceae	norank_f__Oscillospiraceae
FOS	3.34	Mycoplasmatota	Mollicutes	Anaeroplasmatales	Anaeroplasmataceae	Anaeroplasma
FOS	3.19	Firmicutes	Clostridia	Eubacteriales	Eubacteriales incertae sedis	Candidatus_Soleaferrea
FOS	3.18	Firmicutes	Clostridia	Eubacteriales	Oscillospiraceae	Oscillibacter
FOS	3.17	Firmicutes	--	--	--	GCA-900066575
FOS	3.13	Firmicutes	Erysipelotrichia	Erysipelotrichales	Erysipelotrichaceae	Candidatus_Stoquefichus
FOS	3.04	Firmicutes	Erysipelotrichia	Erysipelotrichales	Coprobacillaceae	Erysipelatoclostridium
FOS	3.02	Firmicutes	Clostridia	Eubacteriales	Clostridiaceae	Butyricicoccus
Levan	4.75	Bacteroidota	Bacteroidia	Bacteroidales	Muribaculaceae	norank_f__Muribaculaceae
Levan	4.46	Firmicutes	Erysipelotrichia	Erysipelotrichales	Erysipelotrichaceae	Allobaculum
Levan	4.45	Firmicutes	Erysipelotrichia	Erysipelotrichales	Erysipelotrichaceae	Dubosiella
Levan	4.23	Firmicutes	Clostridia	Eubacteriales	Peptostreptococcaceae	Romboutsia
Levan	3.92	Bacteroidota	Bacteroidia	Bacteroidales	Prevotellaceae	Prevotellaceae_UCG-001
Levan	3.74	Actinomycetota	Actinomycetes	Bifidobacteriales	Bifidobacteriaceae	Bifidobacterium
Levan	3.74	Firmicutes	Bacilli	Lactobacillales	Lactobacillaceae	Lactobacillus
Levan	3.72	Bacteroidota	Bacteroidia	Bacteroidales	Rikenellaceae	Alistipes
Levan	3.59	Bacteroidota	Bacteroidia	Bacteroidales	Tannerellaceae	Parabacteroides
Levan	3.35	Firmicutes	Clostridia	Eubacteriales	Lachnospiraceae	Ruminococcus_torques_group
Levan	3.34	Pseudomonadota	Betaproteobacteria	Burkholderiales	Sutterellaceae	Parasutterella
Levan	3.28	Firmicutes	Bacilli	Bacillales	Bacillaceae	Bacillus
Levan	3.25	Firmicutes	--	--	--	unclassified_p__Firmicutes
Levan	3.23	Firmicutes	Erysipelotrichia	Erysipelotrichales	Erysipelotrichaceae	Faecalibaculum

Supplementary Table 2. Antibody information of immunofluorescence staining

Antibody	Catalog	Supplier	Dilution
ZO-1	GB111981	Servicebio	1: 500
Vimentin	GB11192	Servicebio	1: 100
Mucin2	GB11344	Servicebio	1: 500
Chromogranin A	GB111316	Servicebio	1: 1500
CY3-labeled goat anti-rabbit IgG	GB21303	Servicebio	1:300

Supplementary Table 3. Number of valid sequences per sample in 16s rRNA gene sequencing.

Sample	ASV_num	Seq_num	Mean±SD of Seq_num
FOS1	461	128609	
FOS2	499	122357	
FOS3	491	137234	130385.2±5699.9
FOS4	471	134216	
FOS5	488	129510	
Levan1	550	115706	
Levan2	458	129327	
Levan3	605	125419	120860.6±16026.7
Levan4	443	95980	
Levan5	477	137871	
C1	543	107678	
C2	575	104352	
C3	427	105121	111437.2±11200.8
C7	529	131206	
C8	494	108829	
D1	583	137662	
D2	472	119025	
D3	472	109598	115859.2±16362.1
D4	459	92880	
D5	474	120131	

Supplementary Table 4. Number of clean reads per sample in metagenomic sequencing.

Samples	Clean reads	Percent in raw reads (%)	Mean±SD of clean reads
Con_Dub1	47007706	97.93239439	
Con_Dub2	44542614	97.66677377	
Con_Dub3	43133802	97.47992866	47575042.8±5547887.3
Con_Dub4	46048240	96.54503177	
Con_Dub5	57142852	96.24573135	
Con1	43447370	96.00495765	
Con2	45666082	96.58039276	
Con3	44585442	97.17792523	46161723.2±2644269.2
Con4	46763718	96.02330051	
Con5	50346004	97.00486099	
DSS_Dub1	50202262	98.07231722	
DSS_Dub2	47796618	98.44952449	
DSS_Dub3	47153112	97.04194911	49366481.2±1932311.4
DSS_Dub4	51964834	97.94548857	
DSS_Dub5	49715580	97.52872949	
DSS1	42419126	97.718203	
DSS2	47546152	97.84535528	
DSS3	47651556	96.81591095	45485566.0±2378384.2
DSS4	46249616	98.38504239	
DSS5	43561380	97.28513831	
